# The association between CT-assessed sarcopenic overweight, obesity and rupture abdominal aortic aneurysm: a cross-sectional analysis

**DOI:** 10.3389/fnut.2026.1851995

**Published:** 2026-05-26

**Authors:** Jiashu Yao, Yepeng Zhang, Jie Wang, Wenqing Chen, Xiangrui Li, Guangyan Wu, Min Zhou, Zhonghua Li, Bo Gao, Xiaotian Chen

**Affiliations:** 1 Department of Clinical Nutrition, Nanjing Drum Tower Hospital, The Affiliated Hospital of Nanjing University Medical School, Nanjing, Jiangsu, China; 2 Department of Vascular Surgery, Nanjing Drum Tower Hospital, The Affiliated Hospital of Nanjing University Medical School, Nanjing, China; 3 Department of Vascular Surgery, The Affiliated Yixing Hospital of Jiangsu University (Yixing People’s Hospital), Yixing, Jiangsu, China

**Keywords:** abdominal aortic aneurysm, obesity, overweight, rupture, sarcopenia

## Abstract

**Objective:**

To investigate the associations of sarcopenic overweight and obesity with ruptured abdominal aortic aneurysm (rAAA).

**Methods:**

This cross-sectional study retrospectively enrolled 244 patients with abdominal aortic aneurysm (AAA) between January 2022 and December 2024 (mean age: 71.09 ± 10.59 years, mean BMI: 23.39 ± 3.40 kg/m^2^, men: 80.74%). Baseline clinical characteristics, computed tomography (CT) parameters, and laboratory results were collected. Sarcopenia was diagnosed using CT measured skeletal muscle index (SMI) measured at the third lumbar vertebral level (L3). Overweight and obesity were defined according to the World Health Organization (WHO) Asia-Pacific criteria (overweight: BMI ≥ 23 kg/m^2^; obesity: BMI ≥ 25 kg/m^2^). Patients were stratified into two group: non-ruptured AAA group (*n* = 191) and ruptured AAA group (*n* = 53). The associations of sarcopenia, overweight, obesity, and their interaction with rAAA were examined using two-factor logistic regression models.

**Results:**

Neither sarcopenia nor overweight alone showed a significant independent association with rAAA. Obesity was significantly associated with rAAA (odds ratio [OR] 2.15, 95% confidence interval [CI] 1.13 ~ 4.08, *p* = 0.020). In the two-factor logistic regression model, the interaction between sarcopenia and overweight was significantly associated with rAAA (OR = 3.86; 95% CI: 1.30 ~ 11.45; *p* = 0.015). A similar interaction was observed for sarcopenia and obesity (OR = 4.91; 95% CI: 1.57 ~ 15.34; *p* = 0.006).

**Conclusion:**

Sarcopenic overweight and obesity were independently associated with rAAA. The coexistence of low muscle mass and excess adiposity, rather than either condition alone, was associated with higher odds of rupture. Integrating CT-derived skeletal muscle assessment with BMI may help identify patients with higher odds of rupture.

## 1 Introduction

Abdominal aortic aneurysm (AAA) is an asymptomatic, and progressive vascular disorder affecting millions of individuals globally each year (1). It is characterized by gradual aortic dilation, and the risk of rupture rises as the aneurysm diameter increases, particularly when exceeding 5.5 cm. Rupture AAA carries an extremely high mortality rate with up to 70–80% of patients dying before reaching the hospital (2). Even among those who undergo emergency surgical or endovascular repair, the in-hospital mortality rate remains at 20–40% (3, 4). Critically, approximately 50% of patients with ruptured AAA fail to survive until definitive intervention, highlighting the urgent need for biomarkers and integrated risk assessment strategies (5).

Established clinical risk factors for AAA progression and rupture include male sex, advanced age, hypertension, smoking, and aneurysm diameter (1, 6). To facilitate risk stratification of AAA patients and develop rupture risk prediction models, an increasing number of studies have integrated multi-dimensional data. Such data encompass clinical characteristics, laboratory parameters, aneurysm morphological features, and biomechanical properties (1, 6–8). Nevertheless, the predictive performance of these models in real-world clinical practice remains to be fully validated. Moreover, their generalizability across diverse ethnic populations remains unclear, which limits their application in clinical settings across different geographic regions.

Sarcopenic obesity is a clinical syndrome characterized by the loss of skeletal muscle mass and function, along with abnormal adipose tissue accumulation (9). This condition exhibits an age-related increase in prevalence, with a global prevalence of up to 11% in individuals aged ≥ 60 years (10), and is even more prevalent in elderly inpatients (11). Consequently, it has emerged as a major global concern in geriatric health. Accumulating evidence indicates that sarcopenic obesity is closely positively associated with an elevated risk of multiple adverse health outcomes, including cardiovascular diseases, diabetes mellitus (DM), stroke, and malnutrition (12, 13). Furthermore, it substantially elevates all-cause and cardiovascular mortality, posing a severe threat to population health worldwide (11, 12).

While emerging evidence suggests an important role for sarcopenia in AAA, existing research has largely focused on its association with postoperative outcomes. A strong association has been established between sarcopenia and adverse postoperative outcomes in AAA, including elevated mortality risk and increased complication rates (14–17). Furthermore, the prevalence of sarcopenia is notably higher among AAA patients undergoing endovascular repair, with a prevalence rate of up to 67% (18).

Despite these established associations, it remains unknown how sarcopenic overweight and obesity influences the natural history of AAA, particularly with respect to rupture occurrence. This study therefore aims to define their relationships with rAAA, thereby providing evidence to refine clinical stratification and guide personalized management in this patient population.

## 2 Methods

### 2.1 Study design and participants

**Figure 1:**
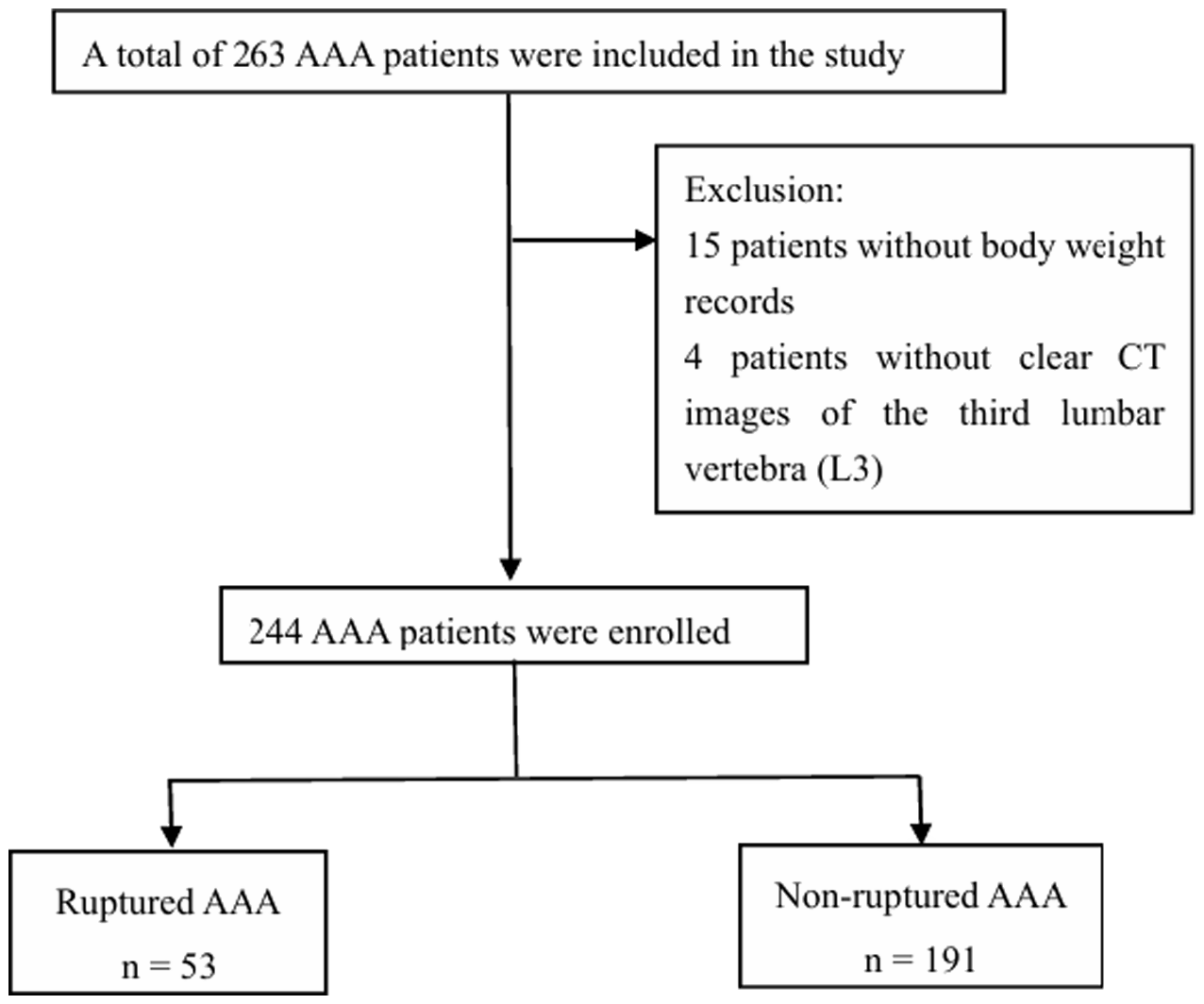
Flow chart of participant selection.

This was a single-center, retrospective cross-sectional comparative study conducted at Nanjing Drum Tower Hospital, affiliated with Nanjing University School of Medicine. Clinical data of patients diagnosed with AAA and admitted to our hospital between January 2022 and December 2024 were collected. Initially, 263 patients with AAA were enrolled. According to predefined exclusion criteria, 15 patients lacking admission body weight records and 4 patients with unclear computed tomography (CT) images at the third lumbar vertebra (L3) level were excluded. Finally, a total of 244 eligible patients were included for subsequent analyses (Figure 1). Based on the presence or absence of aneurysm rupture, patients were stratified into two groups: the non-ruptured AAA group (*n* = 191) and the ruptured AAA group (*n* = 53). This study was conducted in strict compliance with the ethical principles of the Declaration of Helsinki and was approved by the Ethics Committee of Nanjing Drum Tower Hospital, Affiliated Hospital of Nanjing University Medical School (approval number: 2022-086-02). Written informed consent was obtained from all enrolled patients or their legal representatives prior to data collection. For patients unable to provide informed consent independently and with legal representatives unavailable to sign on their behalf, a waiver of informed consent was granted by the aforementioned Ethics Committee.

### 2.2 Data collection

Demographic characteristics comprising age and gender, weight, height and body mass index (BMI) were collected from all study participants. Hematological indices within 24 h of admission, such as coagulation profiles, lipid profiles, renal function parameters, fasting plasma glucose (FPG) and C-reactive protein (CRP), were also recorded. Additionally, smoking history and comorbidities were documented in detail.

Previous studies have confirmed that single-slice scanning at the L3 vertebral level is the optimal compromise site for evaluating whole-body skeletal muscle, visceral adipose tissue, and subcutaneous adipose tissue (19–21). Furthermore, this level is regarded as the gold standard for quantitative assessment of trunk musculature (22) and recommended for the diagnostic evaluation of sarcopenia (23). DICOM-format CT images were processed for skeletal muscle area (SMA) segmentation using Slice-O-Matic 5.0 software (TomoVision, Canada). The segmented skeletal muscles included the rectus abdominis, internal and external oblique muscles, transversus abdominis, erector spinae, quadratus lumborum, and psoas major muscles. This software enables delineation of specific tissues via preset Hounsfield unit (HU) thresholds. The CT HU thresholds for each tissue were set as follows: skeletal muscle (−29 to +150 HU), subcutaneous adipose tissue (−190 to −30 HU), and visceral adipose tissue (−150 to −50 HU). The software automatically calculated the cross-sectional area of skeletal muscle (SMA, unit: cm^2^) and skeletal muscle density (SMD, unit: HU). The skeletal muscle index (SMI) was calculated as: SMI = SMA (cm^2^) / height^2^ (m^2^). Similarly, the cross-sectional areas of subcutaneous adipose tissue (SAT) and visceral adipose tissue (VAT) were normalized against height squared (m^2^), yielding SAT and VAT index (unit: cm^2^/m^2^) (24, 25). All tissue cross-sectional areas were automatically computed by the software, followed by manual correction to ensure segmentation accuracy. All CT images were independently analyzed by two systematically trained observers.

### 2.3 Assessment of overweight, obesity and sarcopenia

The diagnosis of sarcopenia referred to the Chinese population research standards, and the specific diagnostic thresholds were as follows: SMI < 39 cm^2^/m^2^ (male) and < 31.1 cm^2^/m^2^ (female) for BMI < 25 kg^2^/m^2^; adjusted thresholds of < 46.2 cm^2^/m^2^ (male) and < 34.2 cm^2^/m^2^ (female) for BMI ≥ 25 kg^2^/m^2^ (20).

Overweight and obesity were defined according to the World Health Organization (WHO) Asia-Pacific criteria (26): overweight was defined as BMI ≥ 23.0 kg/m^2^, and obesity as BMI ≥ 25.0 kg/m^2^.

### 2.4 Statistical analysis

Quantitative data were presented as mean ± standard deviation (Mean ± SD) for normally distributed data and median (interquartile range) for non-normally distributed data. Categorical variables were expressed as frequency (percentages). Intergroup comparisons performed using the t-test or Mann–Whitney U test where appropriate.

The associations of sarcopenia (yes/no), overweight (BMI ≥ 23 kg/m^2^, yes/no) or obesity (BMI ≥ 25 kg/m^2^, yes/no), and their interaction with rAAA were evaluated using a two-factor logistic regression model with an interaction term. Multivariable logistic regression was performed to evaluate the association between sarcopenia, overweight, obesity and rAAA. Three models were constructed: (1) an unadjusted model; (2) Model 1, adjusted for age and sex; and (3) Model 2 (primary adjusted model), further adjusted for AAA diameter and current smoking status. Covariate selection for Model 2 followed two rigorous criteria to ensure model minimize overfitting. This approach was essential given the limited number of observed rupture events (*n* = 53). First, we used a directed acyclic graph (DAG) to identify the minimal sufficient adjustment set based on established AAA pathophysiology (6, 27, 28) (Supplementary Figure S1). Second, we adhered to the events per variable (EPV) rule, which recommends at least 10 events per candidate predictor to maintain the stability of regression coefficients (29). This analysis was performed separately for the overweight definition (BMI ≥ 23 kg/m^2^) and the obesity definition (BMI ≥ 25 kg/m^2^).

To assess the consistency of these associations, exploratory subgroup analyses were performed based on primary clinical risk factors, including sex (male vs. female), age (< 75 vs. ≥ 75 years), AAA diameter (< 5.5 vs. ≥ 5.5 cm), and current smoking (yes vs. no).

After applying exclusion criteria, all 244 patients had complete data for demographic variables, BMI, and CT-derived body composition parameters. Missing data for laboratory covariates were minimal: AAA diameter (2 patients, 0.8%), current smoking status (1 patient, 0.4%). Given the very low proportion of missing data (< 1% for any variable), primary analyses used complete cases. To assess robustness, multiple imputation was performed as a sensitivity analysis (Supplementary Table S1).

Statistical analyses were performed using SPSS 25.0 (SPSS Inc., Chicago, IL, USA) and R, version 4.3.1 (R Project for Statistical Computing). Two-sided *p* < 0.05 was considered statistically significant for all hypothesis tests.

## 3 Results

### 3.1 Characteristics of study population

Table 1 presents the baseline demographic, anthropometric, clinical, and laboratory characteristics of the patients. The overall cohort had a mean age of 71.1 ± 10.6 years and was predominantly male (80.7%). Ruptured AAA was observed in 53 patients (21.7%), and 81.1% of these cases occurred in male patients.

**Table 1:** Basic characteristics of the study population.

Variables	Total (*n* = 244)	Non-ruptured AAA (*n* = 191)	Ruptured AAA (*n* = 53)	*p*
Age, years	71.09 ± 10.59	71.65 ± 9.80	69.09 ± 12.95	0.120^†^
Men, *n* (%)	197 (80.74)	154 (80.63)	43 (81.13)	0.934*
Body weight, kg	66.60 ± 11.79	65.87 ± 11.44	69.23 ± 12.74	0.067^†^
Overweight, *n* (%)	133 (54.51)	102 (53.40)	31 (58.49)	0.511*
Obesity, *n* (%)	72 (29.51)	49 (25.65)	23 (43.40)	0.012*
Height, cm	168.48 ± 7.56	168.47 ± 7.73	168.53 ± 6.95	0.958^†^
BMI, kg/m^2^	23.39 ± 3.40	23.13 ± 3.22	24.31 ± 3.85	**0.024** ^†^
Length of stay, d	12.00 (8.00, 17.00)	12.00 (9.00, 16.50)	9.00 (5.00, 17.00)	0.024*
SMA, cm^2^	133.67 ± 27.43	133.11 ± 27.07	135.66 ± 28.89	0.550^†^
SMD, HU	33.58 ± 7.56	34.63 ± 7.40	29.81 ± 6.96	**<0.001** ^†^
SMI, cm^2^/m^2^	46.93 ± 8.51	46.72 ± 8.22	47.67 ± 9.53	0.472^†^
VATA, cm^2^	122.96 ± 64.56	125.05 ± 67.48	115.45 ± 52.55	0.340^†^
VAT index, cm^2^/m^2^	43.32 ± 22.69	44.02 ± 23.69	40.79 ± 18.65	0.360^†^
SATA, cm^2^	133.67 ± 58.18	134.93 ± 59.21	129.11 ± 54.63	0.521^†^
SAT index, cm^2^/m^2^	47.58 ± 22.13	47.86 ± 22.11	46.33 ± 22.36	0.656^†^
Sarcopenia, *n* (%)	40 (16.39)	29 (15.18)	11 (20.75)	0.332*
TC, mg/dL	152.92 ± 41.76	159.39 ± 39.85	122.19 ± 37.02	**<0.001** ^†^
TG, mg/dL	105.61 (80.32, 153.09)	106.50 (80.76, 155.76)	96.74 (79.88, 125.14)	0.242*
Scr, mg/dL	79.00 (65.00, 111.00)	75.00 (63.50, 91.00)	116.00 (72.75, 165.75)	<0.001*
eGFR, ml/min/1.73m^2^	86.80 (58.05, 108.28)	90.70 (71.75, 110.05)	55.30 (37.00, 91.15)	<0.001*
FPG, mg/dL	5.11 (4.40, 6.52)	4.79 (4.35, 5.79)	7.29 (6.10, 8.54)	<0.001*
CRP, mg/dL	6.80 (3.30, 33.20)	5.40 (3.02, 17.53)	49.50 (19.35, 92.45)	<0.001*
D-Dimer, mg/L	3.35 (1.29, 9.11)	2.72 (1.21, 6.25)	11.20 (5.29, 20.20)	<0.001*
AAA diameter, cm	5.20 (4.30, 7.00)	4.90 (4.00, 6.30)	7.72 (5.42, 9.30)	<0.001*
Current smoking, *n* (%)	55 (22.63)	42 (21.99)	13 (25.00)	0.646*
DM, *n* (%)	54 (22.13)	49 (25.65)	5 (9.43)	0.012*
CKD, *n* (%)	37 (15.16)	29 (15.18)	8 (15.09)	0.987*
CAD, *n* (%)	61 (25.00)	49 (25.65)	12 (22.64)	0.654*
Stroke, *n* (%)	46 (18.85)	42 (21.99)	4 (7.55)	0.017*
PAD, *n* (%)	14 (5.74)	13 (6.81)	1 (1.89)	0.304*

Data are expressed as median [25, 75%], Mean ± SD, or number (%).

^*^Non-parametric Manne Whitney U test (non-normal data).

^†^Student’s t test (normal data).

BMI, body mass index; FPG, fasting plasma glucose; TC, total cholesterol; TG, triglyceride; CRP, C-reactive protein; Scr, serum creatinine concentration; eGFR, estimated glomerular filtration rate; DM, diabetes mellitus (DM); CKD, chronic kidney disease; CAD, coronary heart disease; PAD, peripheral arterial disease; SMA, skeletal muscle mass area; SMD, skeletal muscle mass density; SMI: skeletal muscle index; VATA, visceral adipose tissue area; SATA, subcutaneous adipose tissue area. The bold entries are statistical significance. *p* ≤ 0.05 are statistical significance.

Compared with the non-ruptured AAA group, patients with rAAA exhibited significantly higher prevalence of obesity, along with significantly elevated BMI. SMD was markedly lower in the rAAA group. Hospital length of stay was significantly shorter in the rAAA group. In terms of laboratory parameters, levels of TC and eGFR were significantly reduced in the ruptured group, whereas serum creatinine (Scr), FPG, CRP, and D-dimer levels were significantly elevated. AAA diameter was substantially larger in the rAAA group. The prevalence of DM and prior stroke was significantly lower in the rAAA group (all *p* < 0.05).

No statistically significant differences were observed between the two groups in age, gender, body weight, overweight ratio, height, SMA, SMI, visceral adipose tissue area (VATA), VAT index, subcutaneous adipose tissue area (SATA), SAT index, sarcopenia prevalence, triglyceride (TG) levels, smoking history, or the prevalence of CKD, CAD, and PAD (all *p* > 0.05).

### 3.2 Association between sarcopenia, overweight and rAAA

Logistic regression analyses revealed sarcopenia alone was not significantly associated with rAAA (OR 1.57, 95% CI 0.69 ~ 3.57, *p* = 0.282), and overweight alone was not significant (OR 1.23, 95% CI 0.66 ~ 2.30, *p* = 0.521). In the fully adjusted Model 2, the association persisted for the interaction of sarcopenia and overweight (OR 3.86, 95% CI 1.30 ~ 11.45, *p* = 0.015; Table 2). This interaction was not significantly modified by sex, age, AAA diameter, or current smoking (all *P* for interaction > 0.05; Supplementary Figures S2). To assess the robustness of our findings, sensitivity analyses were performed on the imputed dataset; the results remained largely unchanged (Supplementary Table S1).

**Table 2:** Logistic regression analysis of sarcopenia, overweight, obesity, and their interaction with ruptured abdominal aortic aneurysm.

Variables	Unadjusted OR (95% CI)	*p*	Model 1 OR (95% CI)	*p*	Model 2 OR (95% CI)	*p*
Main effects						
Sarcopenia (ref: no sarcopenia)	1.47 (0.68 ~ 3.17)	0.334	1.72 (0.77 ~ 3.83)	0.186	1.57 (0.69 ~ 3.57)	0.282
Overweight (ref: normal weight)	1.23 (0.66 ~ 2.28)	0.511	1.19 (0.64 ~ 2.20)	0.592	1.23 (0.66 ~ 2.30)	0.521
Obesity (ref: no obesity)	2.22 (1.18 ~ 4.18)	**0.013**	2.13 (1.13 ~ 4.04)	**0.020**	2.15 (1.13 ~ 4.08)	**0.020**
Interaction effect						
Sarcopenia × Overweight	3.08 (1.09 ~ 8.70)	**0.034**	3.86 (1.31 ~ 11.40)	**0.014**	3.86 (1.30 ~ 11.45)	**0.015**
Sarcopenia × Obesity	4.00 (1.34 ~ 11.97)	**0.013**	4.93 (1.59 ~ 15.33)	0.**006**	4.91 (1.57 ~ 15.34)	**0.006**

OR: odds ratio OR, odds ratio; CI, confidential intervals.

Model 1 was adjusted for age and sex.

Model 2 was adjusted for the minimal sufficient structural set derived from the Directed Acyclic Graph (DAG) analysis: age, sex, current smoking status, and AAA diameter.

### 3.3 Association between sarcopenia, obesity and rAAA

In Table 2, obesity alone was significantly associated with rAAA (OR 2.15, 95% CI 1.13 ~ 4.08, *p* = 0.020), whereas sarcopenia alone was not (OR 1.57, 95% CI 0.69 ~ 3.57, *p* = 0.282). Similarly, the interaction between sarcopenia and obesity was statistically significant in the fully adjusted model (OR 4.91; 95% CI: 1.57 ~ 15.34; *p* = 0.006). Exploratory subgroup analyses revealed no significant effect modification by sex, age, AAA diameter, or current smoking status (all *P* for interaction > 0.05; Supplementary Figures S3). These findings remained robust in sensitivity analyses utilizing the imputed dataset (Supplementary Table S1).

## 4 Discussion

Our study provided novel evidence that sarcopenic overweight and sarcopenic obesity were associated with higher odds of rAAA. Importantly, the joint effect of low skeletal muscle mass and excess adiposity appeared stronger than either factor alone in the DAG-guided two-factor model, suggesting a potential synergistic interaction. These associations persisted after adjustment for age, sex, smoking, and AAA diameter, and sensitivity analyses using multiple imputation yielded consistent results. Therefore, rational dietary interventions and moderate physical activity not only contribute to weight reduction but also enhance muscle mass and strength, thereby potentially retarding AAA progression and reducing the odds of rupture.

Accumulating evidence had demonstrated that obesity exerts a pivotal role in the pathogenesis and progression of AAA. Weight gain is strongly associated with increased AAA incidence and AAA-related mortality (30). Obesity notably elevates AAA risk, and numerous clinical studies have corroborated that the severity of obesity in patients correlates positively with abdominal aortic diameter (30–33).

In overweight or obese states, adipose tissue accumulates excessively. This is especially evident in VAT and perivascular adipose tissue (PVAT) (34). Excessive adipose accumulation induces increased reactive oxygen species (ROS) generation, which elicits oxidative stress. Subsequently, a cascade of proinflammatory cytokines [e.g., tumor necrosis factor-*α* (TNF-α), interleukin-6 (IL-6), monocyte chemotactic protein 1, (MCP-1)] is released. These pathological processes promote endothelial dysfunction, accelerate endothelial cell apoptosis, impair vascular repair capacity, and promote vascular sclerosis, both of which are key hallmarks of vascular aging, ultimately contributing to AAA initiation and progression (30, 35). Furthermore, leptin, an adipokine secreted by PVAT, can further induce the production of cytokines such as TNF-α, IL-6, and IL-12 (36). This not only aggravates endothelial dysfunction but also induces vascular remodeling, with this effect being closely associated with AAA diameter (30, 37, 38), which in turn is significantly related to the progression and rupture rate of AAA. Consistent with prior studies, our analysis confirms that both the prevalence of obesity and mean BMI are significantly higher in patients with ruptured AAA compared with those with non-ruptured AAA.

Notably, obesity frequently coexists with sarcopenia, and their co-occurrence—referred to as sarcopenic obesity—exerts synergistic detrimental effects that exceed the simple summation of individual risks. Sarcopenia is recognized as a chronic systemic inflammatory state, and sarcopenic obesity is closely associated with muscle mitochondrial dysfunction. Specifically, sarcopenic obesity can induce ROS production via oxidative stress, leading to elevated levels of pro-inflammatory cytokines including CRP, IL-1, IL-6, TNF-*α* in the absence of infection. These inflammatory responses subsequently impair vascular endothelial function and contribute to adverse health outcomes, including CVD (39–41). Additionally, excessive adipokine secretion and visceral fat accumulation can antagonize the effects of anabolic myogenic factors through pro-inflammatory activities, thereby accelerating muscle mass loss and establishing a vicious cycle of “inflammation-muscle wasting” (41, 42).

Existing evidence has established that sarcopenia is closely linked to postoperative complications and prognosis in patients with AAA (18, 43). AAA patients with sarcopenia exhibit significantly elevated all-cause mortality, as well as increased risks of postoperative spinal cord ischemia and acute kidney injury (17, 44, 45). However, to date, there is still a paucity of studies exploring the association between sarcopenic obesity and rAAA. Given shared pathophysiological underpinnings including chronic inflammation, oxidative stress, endothelial dysfunction and vascular stiffening, suggests that sarcopenic obesity may represent a previously underappreciated yet biologically plausible determinant of AAA instability and rupture.

Our findings demonstrated that the proportion of obesity was significantly increased in the rAAA group. The proportion of overweight had also shown an upward trend, while the incidence of sarcopenia had shown a downward trend, although no significant inter group differences were observed. In the adjusted models, obesity alone was significantly associated with rAAA (OR 2.15, 95% CI 1.13 ~ 4.08, *p* = 0.020), whereas overweight alone (OR 1.23, 95% CI 0.66 ~ 2.30, *p* = 0.521) and sarcopenia alone (OR 1.57, 95% CI 0.69 ~ 3.57, *p* = 0.282) were not. The strongest associations emerged from the interaction terms: sarcopenic overweight (OR 3.86, 95% CI 1.30 ~ 11.45, *p* = 0.015) and sarcopenic obesity (OR 4.91, 95% CI 1.57 ~ 15.34, *p* = 0.006), indicating that the coexistence of low muscle mass and excess adiposity conferred substantially higher odds of rupture than either condition alone. These findings suggest that clinical assessment of patients with AAA should evaluate sarcopenia and obesity concurrently. Particular attention is warranted for individuals with both reduced muscle mass and elevated BMI. Although BMI measurement is more convenient than direct muscle strength and mass testing, integrating both assessments may improve identification of patients at higher odds of rupture. We recommended that patients with AAA undergo regular weight monitoring and implement tailored weight control strategies. In summary, our study indicated that maintaining a healthy weight and adequate muscle mass in AAA patients may contribute to delaying the progression of AAA and reducing the odds of rupture. This conclusion also offers valuable clinical insights for optimizing the AAA risk stratification system and formulating personalized intervention plans in the future.

In our study, both BMI ≥ 23 kg/m^2^ (overweight) and BMI ≥ 25 kg/m^2^ (obesity), when combined with sarcopenia, were independently positively associated with rAAA. Although the obesity-based definition yielded a numerically larger OR (OR = 4.91) than the overweight-based definition (OR = 3.86), the overlapping confidence intervals prevent any firm conclusion that one cutoff is superior. From a clinical standpoint, BMI ≥ 23 kg/m^2^ may be more appropriate for broad screening, while BMI ≥ 25 kg/m^2^ may be utilized to flag a higher-odds subgroup requiring closer surveillance. Therefore, these two thresholds may be considered complementary in clinical stratification. However, these findings should be interpreted cautiously, and prospective studies are needed to validate the optimal cutoff.

Interestingly, our study showed that, although SMI did not differ significantly between the ruptured and non-ruptured groups, SMD was markedly lower in patients with rAAA. SMD reflects intramuscular fat infiltration (myosteatosis) and is increasingly recognized as an indicator of poor muscle metabolic health (46). Reduced SMD is closely associated with chronic low-grade inflammation, insulin resistance, and oxidative stress (46–49)—pathological processes that are also central to extracellular matrix degradation and aortic wall weakening in AAA (40, 50). This finding suggests that muscle quality may be a more sensitive marker of the systemic inflammatory burden associated with AAA rupture. However, SMD was not included in multivariable modeling, as our primary aim was to evaluate the combined effect of muscle quantity and adiposity. Future studies should incorporate SMD into risk prediction models to assess whether myosteatosis independently augments rupture risk.

This study has several limitations. First, its retrospective, single-center design introduced potential selection bias. Second, the sample size was relatively limited, particularly the sparse number of events in the rAAA group (*n* = 53), which compromises the overall statistical power. Crucially, this limited event rate renders our exploratory subgroup analyses highly susceptible to overfitting and unstable estimations. Therefore, subgroup-specific trends should be interpreted with extreme caution. Larger cohorts are required to robustly evaluate these interactions. Third, we did not systematically collect or adjust for lipid-lowering medication use, particularly statins. This is an important limitation because statins may lower total cholesterol, exert anti-inflammatory effects, and potentially influence aortic wall stability and body composition. Therefore, the observed lower TC level in the rupture group should be interpreted with caution, as it may partly reflect medication exposure rather than an intrinsic difference in lipid metabolism. The absence of medication data may have introduced residual confounding into the observed associations between lipid profiles, adiposity, and rAAA. Fourth, objective measures (e.g., grip strength, gait speed) were not utilized to assess muscle function; sarcopenia diagnosis relied solely on CT-derived SMI. Furthermore, the independent association between SMD and rAAA was not evaluated in multivariable analyses. Fifth, the size and location of AAA may compromise the accuracy of skeletal muscle area measurements at the L3 vertebral level through compression or traction of adjacent tissues. To mitigate this, patients with poor muscle quality were excluded from the present study, and thus our findings may not be generalizable to this specific patient population. Sixth, the cross-sectional design limited causal inference and reverse causation could not be excluded. In the context of AAA rupture, acute stress and systemic inflammation may alter muscle metabolism and fluid distribution. This may bias CT-derived body composition measures. Future prospective, large-scale longitudinal studies are warranted to further validate our findings. Additionally, integrating muscle function indicators into the assessment system will help improve the reliability and generalizability of the results.

## 5 Conclusion

In this study, we retrospectively analyzed the association between sarcopenic overweight/obesity and rAAA. Both sarcopenic overweight and obesity were independently associated with rAAA. Our findings suggested that identify patients with sarcopenic overweight or obesity may enable timely, targeted interventions, including nutritional support, exercise training, and weight management. Such integrated strategies may help reduce rupture risk and improve long-term outcomes in AAA patients.

## Data Availability

The original contributions presented in the study are included in the article/Supplementary material, further inquiries can be directed to the corresponding authors.
