# Supplementary material for: The association between CT-assessed sarcopenic overweight, obesity and rupture abdominal aortic aneurysm: a cross-sectional analysis
Source: Front Nutr. 2026 May 26;13:1851995. doi: 10.3389/fnut.2026.1851995 (PMC13246379; doi:10.3389/fnut.2026.1851995)
Supplement: Supplementary file 1 [file Table_1.DOCX]

**Supplementary Table S1.** Sensitivity analysis: comparison of multiple imputation results for the association of sarcopenia, overweight, obesity, and their interaction with ruptured abdominal aortic aneurysm

|  | Unadjusted  OR (95% CI) | *P* | Model 1  OR (95% CI) | *P* | Model 2  OR (95% CI) | *P* |
| --- | --- | --- | --- | --- | --- | --- |
| **Main effects** |  |  |  |  |  |  |
| Sarcopenia   (ref: no sarcopenia) | 1.47 (0.68 ~ 3.17) | .334 | 1.72 (0.77 ~ 3.83) | .186 | 1.68 (0.74 ~ 3.83) | .219 |
| Overweight  (ref: normal weight) | 1.23 (0.66 ~ 2.28) | .511 | 1.19 (0.64 ~ 2.20) | .592 | 1.15 (0.61 ~ 2.17) | .661 |
| Obesity  (ref: no obesity) | 2.22 (1.18 ~ 4.18) | **.013** | 2.13 (1.13 ~ 4.04) | **.020** | 2.16 (1.13 ~ 4.13) | **.020** |
| **Interaction effect** |  |  |  |  |  |  |
| Sarcopenia × Overweight | 3.08 (1.09 ~ 8.70) | **.034** | 3.86 (1.31 ~ 11.40) | **.014** | 3.91 (1.32 ~ 11.59) | **.014** |
| Sarcopenia × Obesity | 4.00 (1.34 ~ 11.97) | **.013** | 4.93 (1.59 ~ 15.33) | .**006** | 4.99 (1.60 ~ 15.62) | **.006** |

OR: odds ratio OR, odds ratio; CI, confidential intervals;

Model 1 was adjusted for age and sex.

Model 2 was adjusted for the minimal sufficient structural set derived from the Directed Acyclic Graph (DAG) analysis: age, sex, current smoking status, and AAA diameter.


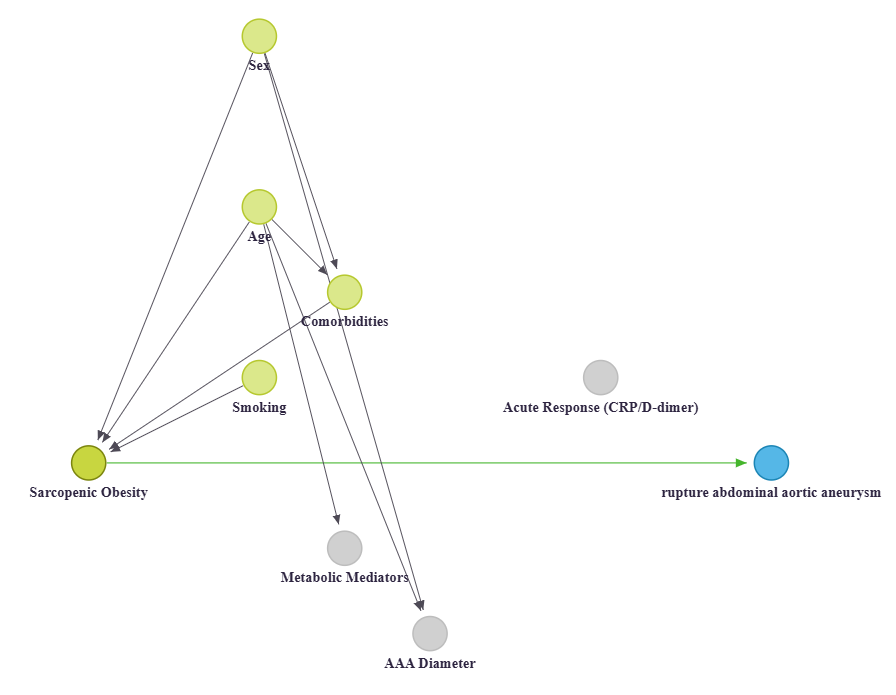


**Supplementary Figure S1.** The directed acyclic graph (DAG) framework based on established AAA pathophysiology.

**
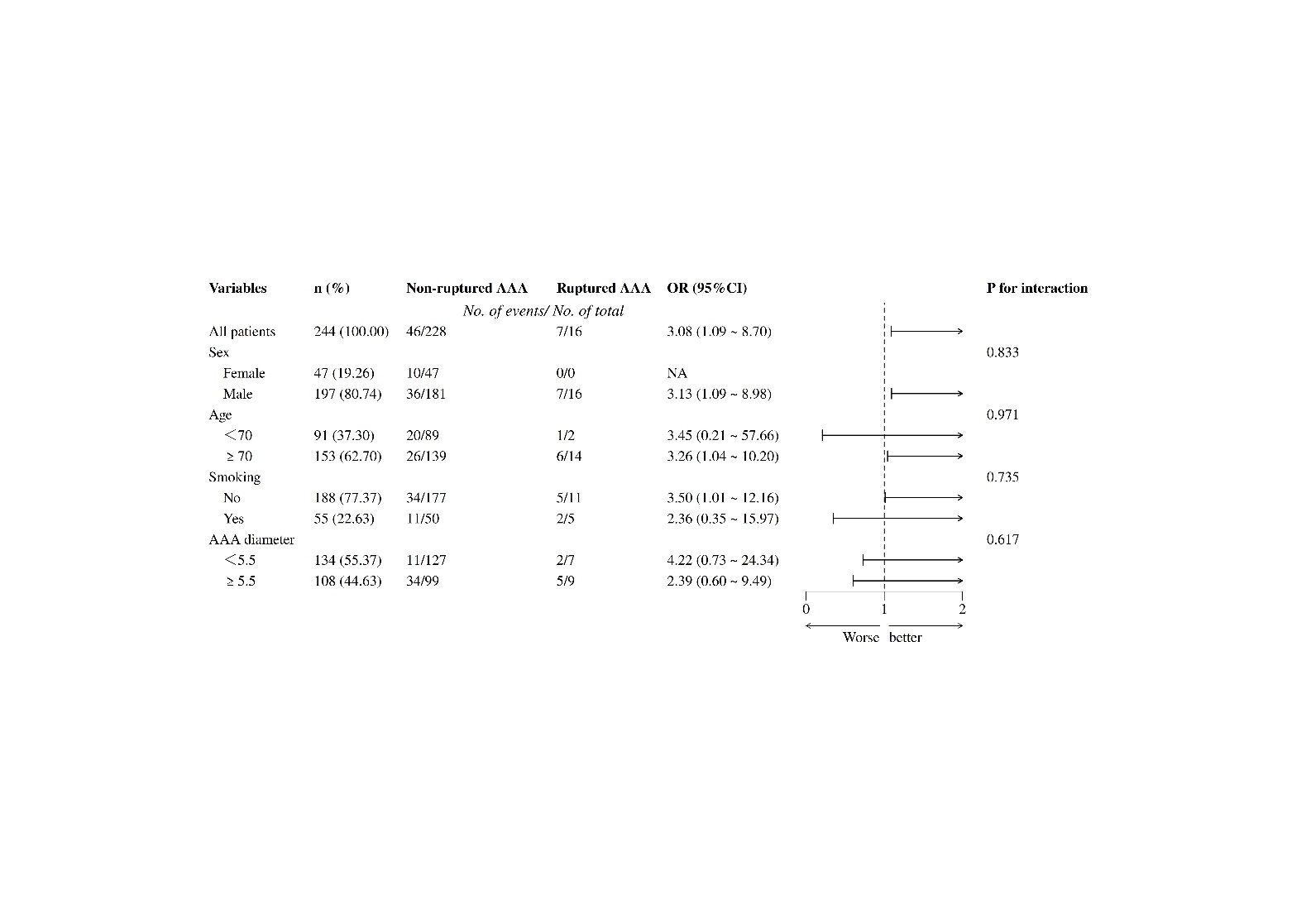
**

**Supplementary Figure S2.** Subgroup analyses of the association between sarcopenic overweight and odds of ruptured abdominal aortic aneurysm. The criteria for subgroup classification have been detailed in the Methods section.

**
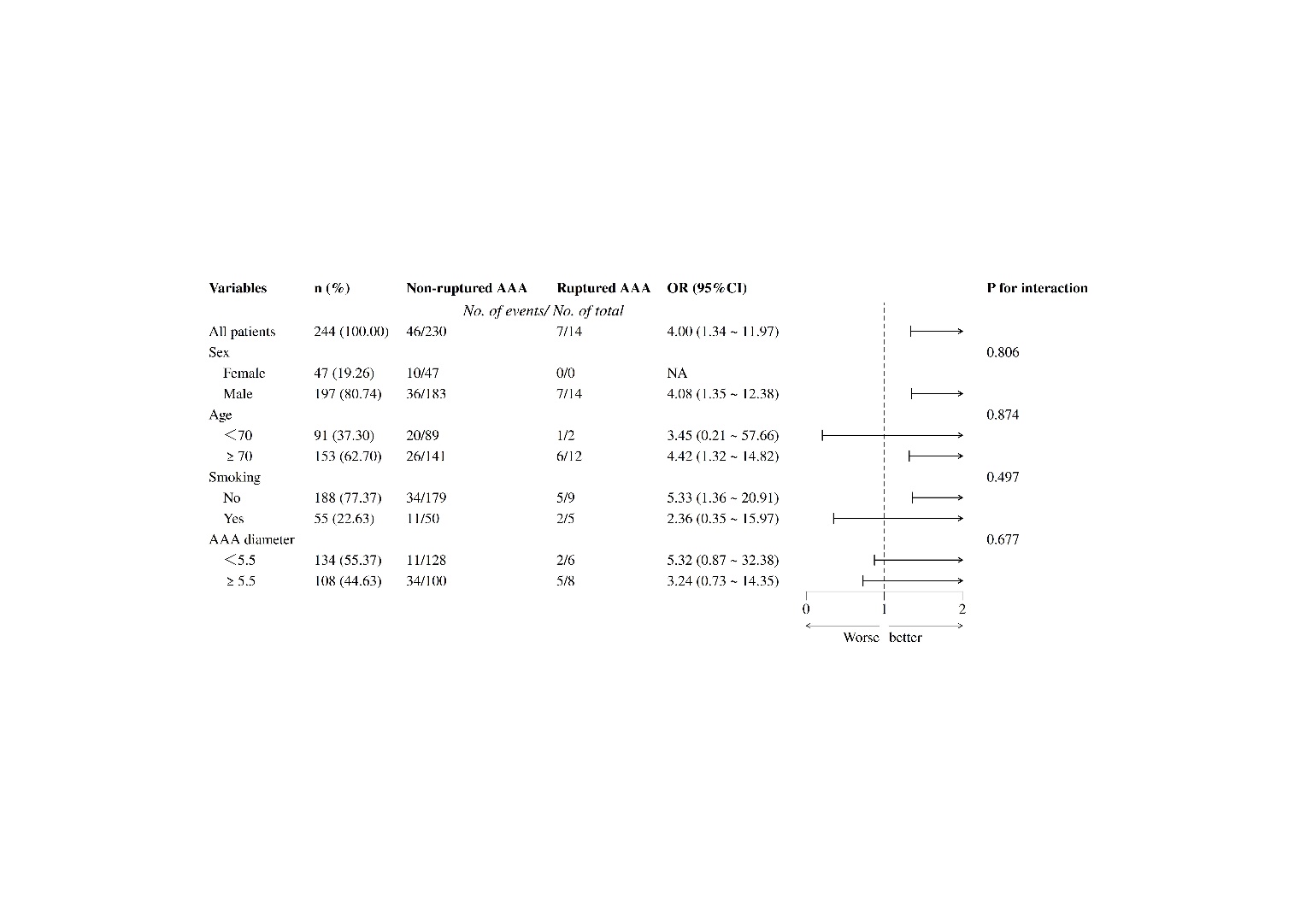
**

**Supplementary Figure S3.** Subgroup analyses of the association between sarcopenic overweight and odds of ruptured abdominal aortic aneurysm. The criteria for subgroup classification have been detailed in the Methods section.
